# “The effect of intramuscular dexmedetomidine versus oral gabapentin premedication on the emergence agitation after rhinoplasty”. A prospective, randomized, double-blind controlled trial

**DOI:** 10.1186/s12871-025-02914-5

**Published:** 2025-01-31

**Authors:** Tamer Samir Abdelsalam Abdelaziz, Hatem Elsayed Mohammed Elsayed, Doaa Mohammed Kamal Eldin, Ismail Mohammed Ibrahim

**Affiliations:** Department of Anesthesia, Intensive Care and Pain Management, Faculty of Medicine, Ain Shams University, Cairo, Egypt

**Keywords:** Dexmedetomidine, Emergence agitation, Gabapentin, General anesthesia, Postoperative pain, Rhinoplasty

## Abstract

**Background:**

Emergence agitation EA is a state of confusion and harmful aggressiveness during recovery. It is a common complication after rhinoplasty, with risk of trauma, bleeding, and hemodynamic instability. Dexmedetomidine and gabapentin premedication could improve the quality of recovery after rhinoplasty.

**Methods:**

One hundred fifty-three participants (ASA I-II, both sexes and age 18–40 years) scheduled for rhinoplasty were randomized into three groups. Group C didn’t receive premedication, Group D received intramuscular (IM) dexmedetomidine, and Group G received oral gabapentin. The degree of EA by Riker sedation-agitation scale (RSAS) during recovery, pain severity, and adverse events recorded.

**Results:**

The results showed statistically significant differences in EA scores with the lowest values in group D (p-value 0.002). Moreover, the incidence of EA was 17.6% (9/51) in Group D, 41.2% (21/51) in Group G, and 56.9% (29/51) in Group C with P value < 0.001 and significant differences in VAS score at 4, 8, and 12 h with the highest median (range) values in group C 4(3–6) in comparison to group D 2(1–3) and group G 2(1–3) and p-value < 0.001; no significant differences in adverse events.

**Conclusions:**

IM dexmedetomidine premedication was more efficient than gabapentin in the reduction of the emergence agitation incidence, severity, and postoperative pain scores after rhinoplasty in adults.

**Clinical trial registration number ID:**

NCT05626998 on 25/11/2022.

## Introduction

Emergence agitation [EA] is a state of confusion and harmful aggressiveness during anesthesia recovery [1]. Trauma, bleeding, tubes and catheter removal, desaturation, and hemodynamic instability were reported [2].

21% of adults suffer from EA. The predisposing factors include sex, age, operation, anesthesia, pain, full bladder, and intubation [3]. The incidence was higher in the pediatric and adult populations following nasal and throat surgeries [1].

Emergence agitation is a well-known complication after rhinoplasty due to posterior nasal discharge, bleeding, pain, and nasal block [2].

Dexmedetomidine, an α2 adreno-receptor agonist, provides analgesia, anxiolysis, and sedation without respiratory depression. High doses and fast intravenous injections of dexmedetomidine in adults caused hypotension and bradycardia [4]. A low-dose intramuscular dexmedetomidine (1 µg/kg) provides sedation and promotes anesthesia without hemodynamic side effects [5]. Moreover, the incidence, duration, and severity of emergence agitation decreased with fewer intraoperative movements [6].

Gabapentin is structurally like the inhibitory transmitter gamma amino-butyric acid (GABA) without the GABA receptor effect. It prevents presynaptic calcium influx through voltage-sensitive channels [7], increases GABA synthesis and concentration, decreases excitatory neurotransmitter release, and blocks stimulus progression in the brain and dorsal root ganglia [8].

Gabapentin has anticonvulsant, anxiolytic, sedative, and analgesic effects. It decreases the pressor response to laryngoscopy and endotracheal intubation, maintains intraoperative hemodynamic stability, and can reduce postoperative delirium and agitation [9].

Gabapentin decreased narcotic requirements and postoperative pain in the previous literature [10]. It also reduced the emergence agitation incidence and severity after mastectomy in adults with limited adverse effects [11].

Statement of Clinical Relevance: There are limited studies concerning adult EA, and although its prevalence is less than child EA, it carries more risk of injury due to serious uncontrolled behaviors. This study assessed and compared the effects of dexmedetomidine and gabapentin premedication on EA incidence and severity after rhinoplasty in adults.

## Materials and methods

A prospective, randomized, and parallel research was registered and approved by the Ethics Committee of the Faculty of Medicine, registered at ClinicalTrials.gov, and conducted according to the Consolidated Standards of Reporting Trials (CONSORT) guidelines in the University Hospital between 1st November 2022 and 30th April 2023. All participants signed written informed consent.

Participants undergoing rhinoplasty who fulfilled the inclusion criteria were randomly randomized to one of the following three groups using computer-generated codes placed in opaque sealed envelopes with a 1:1 ratio by a physician not directly involved in the research or patient care.

Group C (the control group) didn’t receive premedication.

Group D (dexmedetomidine group) received (1 µg/kg) intramuscular dexmedetomidine 30 min before the operation [5].

Group G (gabapentin group) received 600 mg of gabapentin 30 min before the operation by mouth [11].

Clinical pharmacy physicians prepared the study drugs, and a nurse gave them to the participants in the ward. Follow-up was achieved by anesthesiologist residents unaware of group allocation, and they were responsible for assessing the participants’ Riker sedation-agitation scale (RSAS) score. So, the participants, allocating physicians, nurses, and follow-up physicians, were blinded.

The participants who fulfilled the inclusion criteria were involved: ASA I–II, both sexes, 18–40 years old, and undergoing rhinoplasty.

The participant was excluded if he refused to participate or sign the consent, medicated with or was allergic to study drugs, had cardiovascular, hepatic, renal failure, psychiatric (tricyclics or MAOIs), bleeding tendency disorders, or drug and alcohol abuse.

All participants were assessed clinically, and routine preoperative investigations were done, including CBC, coagulation profile, liver function tests, kidney function tests, fasting blood sugar, and ECG.

The participants were informed that post-operative nasal packs and splints might be uncomfortable.

In the operating room, the standard monitoring was connected, and baseline parameters were recorded, including heart rate HR, mean arterial blood pressure MAP, and oxygen saturation Spo2. An intravenous (IV) line was inserted, and the sedation scale (RSAS) was checked before anesthesia.

In all participants, propofol 2 mg/kg, fentanyl 2 µg/kg, and rocuronium 0.6 mg/kg were used for anesthesia induction with oral endotracheal intubation. The surgeon infiltrated the operative site with lignocaine HCL 2% and 1:200,000 adrenaline to reduce bleeding and pain [12].

End-tidal CO2 was monitored, and mechanical ventilation parameters were adjusted to maintain normocapnia. Isoflurane 1–2% was used for anesthesia maintenance [11], and nasal splints were fixed to support and protect the nose at the end of surgery [12].

Isoflurane was discontinued 5 min before surgery completion, and the neuromuscular blockade was antagonized with 2 mg/kg sugammadex at the end of surgery [11, 12].

The participants extubated after regaining spontaneous respiration and adequate muscle power. RSAS score and level of agitation preoperatively and during recovery were assessed by anesthesia residents unaware of group allocation [11].

In the recovery room, a trained nurse monitored the patients every 5 min and recorded the degree of EA using RSAS, HR, SpO2, BP, pain using the VAS score (0 = no pain; 10 = worst possible pain), and nausea & vomiting.

Participants received intravenous pethidine (25–50 mg) for VAS score ≥ 4, and ondansetron (4 mg) for nausea and vomiting. Midazolam (0.02 mg/kg) was given for RSAS ≥ 5 after excluding other causes of agitation such as hypoxia and a distended urinary bladder [11].

Participants were followed up for 1 h in the PACU and transferred to the ward with a modified Aldrete recovery score > 9 and no shivering, bleeding, nausea, or vomiting [11] with a recording of recovery time. One gram of IV paracetamol was prescribed regularly every 8 h, and intravenous pethidine (25–50 mg) was given for VAS score ≥ 4 in the ward.

- **The primary outcome**: Emergence agitation (EA) at anesthesia recovery was assessed by the Riker Sedation-Agitation Scale (from 1 = unarousable to 7 = dangerous agitation, with a score ≥ 5 considered EA).
- **The secondary outcome**:
  - Post-operative pain was assessed by a visual analog scale (0 no pain and 10 worst possible pain) at 4, 8, and 12 h.
  - Adverse events include dizziness, headache, nausea, and vomiting at 12 h.

Using PASS 11 software for sample size calculation and setting power at 80%, alpha error at 5%, and after reviewing previous study results (Azemati et al., 2013) [11] showed that the mean agitation number after recovery among patients underwent breast cancer surgery who took gabapentin was lower than those took a placebo (4.08 ± 0.44 versus 4.40 ± 0.67, respectively); based on that, a sample size of at least 153 patients undergoing rhinoplasty will be divided randomly into 3 groups (51 patients in each group) will be sufficient to achieve the study objective.

Statistical Package for Social Science (SPSS) version 27.0. was used to analyze the study data, Quantitative data were shown as mean ± standard deviation (SD) or median and range. Qualitative data were shown as frequency and percentage. Analysis of variance (ANOVA) was utilized to test the difference between the means of several independent groups, and when it was positive, the post-hoc test for pairwise comparison of subgroups was used. The chi-square was used to show the relationship of the qualitative variables. The Kruskal-Wallis test was used for non-parametric data comparison, and pairwise comparison of subgroups was utilized when the test was positive. A P-value < 0.05 was considered significant.

## Results

**Fig. 1:**
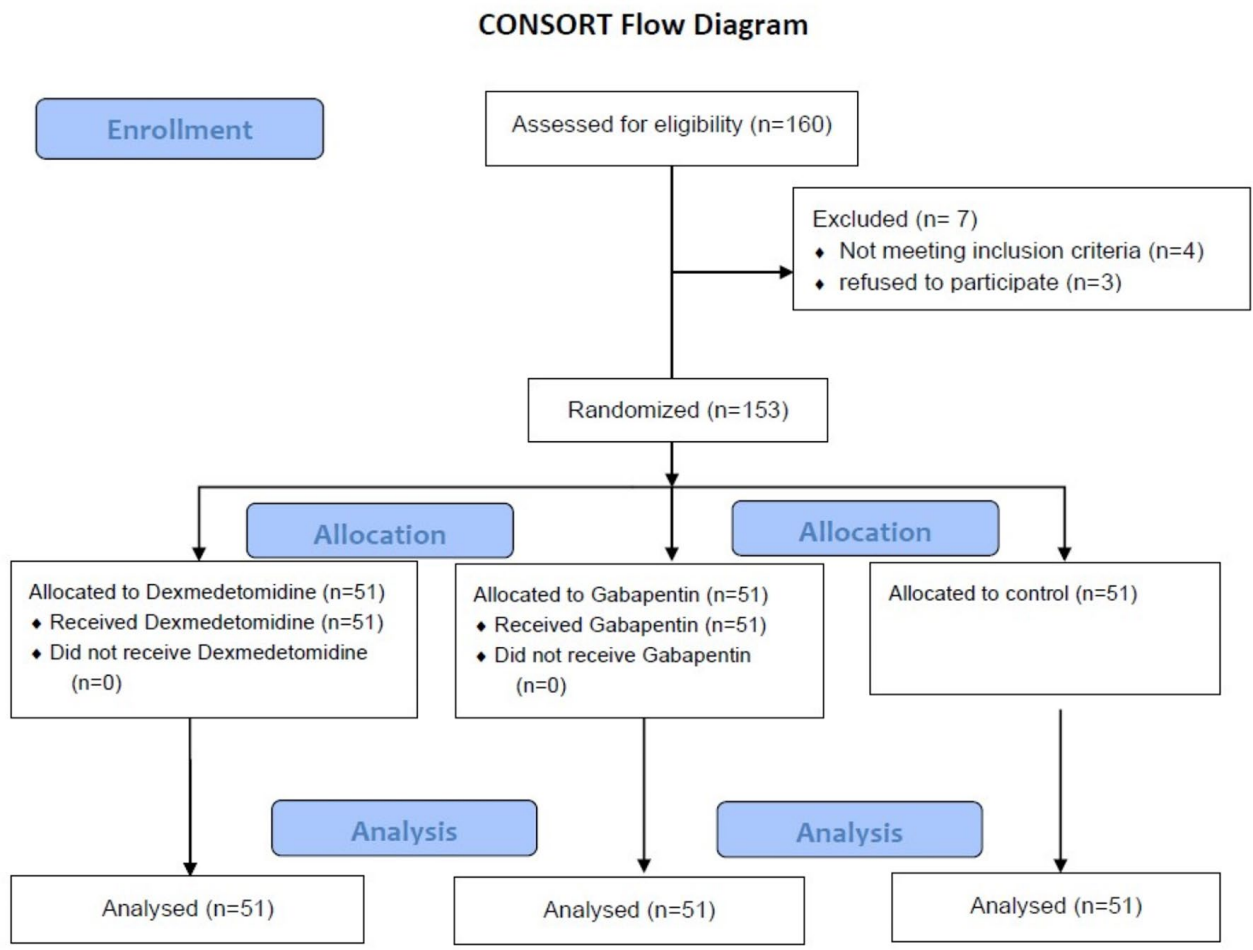
CONSORT Flow Diagram

One hundred sixty participants were screened, and seven participants were excluded (3 refused to participate and 4 did not meet the inclusion criteria). The 153 participants were randomized into three equal groups and were available for the final analysis (Fig. 1).

The results didn’t show significant differences in terms of demographic data (age, sex, weight, height, recovery time, and operative time) and hemodynamics (HR and MAP) at baseline, pre-, intra-, and post-operative parameters between the three groups with a p-value > 0.05 (Table 1).

**Table 1:** Demographic data and hemodynamic parameters

		Group C (*n* = 51)	Group D (*n* = 51)	Group G (*n* = 51)	*p*
Age(years)		30 ± 7.2	29.76 ± 7.3	30.5 ± 7	0.87
Sex	M	28 (54.9%) 23 (45.1%)	26 (51%) 25 (49%)	24 (47.1%) 27 (52.9%)	0.73*
	F				
Weight(kg)		71.8 ± 4.7	72.2 ± 4.7	71.8 ± 5	0.93
Height(cm)		169.5 ± 3.8	169.3 ± 5	170.5 ± 5.3	0.37
Operative time(min)		86.3 ± 14.7	82.4 ± 16.5	84.2 ± 16.6	0.47
Recovery time(min)		51.4 ± 9	51.9 ± 9	50.1 ± 9.8	0.612
HR baseline		92.8 ± 2.3	93.5 ± 2.11	92.4 ± 3.7	0.18
HR preop		87.6 ± 2.3	86.8 ± 2.1	86.8 ± 2.2	0.1
HR intraop		84.2 ± 3.2	83.6 ± 4	83 ± 3.1	0.24
HR postop		87.6 ± 2.4	87.5 ± 2.8	87.2 ± 3	0.76
MAP baseline		98.8 ± 4.2	98.8 ± 5	98.9 ± 5.6	0.97
MAP preop		95.2 ± 4	95.4 ± 3.6	95.9 ± 3.6	0.6
MAP intraop		88.3 ± 3.9	88.5 ± 3.5	88.9 ± 3.6	0.65
MAP postop		93 ± 3.3	92.7 ± 3.7	92.8 ± 3.6	0.85

Data expressed as mean ± SD, one way ANOVA, *= chi square test. p-value > 0.05 is considered statistically non-significant. HR = beat/min and MAP = mmHg

The results showed statistically significant differences in EA scores (p value 0.002) and midazolam consumption (p value 0.01) with the lowest values in Group D; Moreover, the incidence of EA was 17.6% (9/51) in Group D, 41.2% (21/51) in Group G, and 56.9% (29/51) in Group C with a P-value < 0.001 (Table 2).

**Table 2:** Emergence agitation and VAS score

	C	D	G	*P* value
EA	5 (3–7)	4 (2–5)	4 (2–6)	0.002
Incidence of EA(*n* = 51)	29(56.9%)	9(17.6%)	21(41.2%)	< 0.001*
Postoperative pethidine consumption(mg)	93.7 ± 12	33.14 ± 18.6	40.5 ± 21.5	< 0.001#
Midazolam(mg)	1.5(0–4)	0(0–4)	0(0–4)	0.01
VAS 4 h	4 (3–6)	2 (1–3)	2 (1–3)	< 0.001
VAS 8 h	4 (3–5)	2 (1–3)	2 (1–4)	< 0.001
VAS 12 h	4 (3–5)	3 (2–4)	3 (2–4)	< 0.001
Pairwise comparisons				
	D-G	D-C	G-C	
EA	0.006	< 0.001	0.023	
Postoperative pethidine consumption#	0.117	< 0.001	< 0.001	
Midazolam	0.439	0.003	0.03	
VAS 4 h	0.413	< 0.001	< 0.001	
VAS 8 h	0.306	< 0.001	< 0.001	
VAS 12 h	0.391	< 0.001	< 0.001	

Data expressed as median (range), mean ± SD, frequency (percentage). p value = Kruskal-Wallis test, *= chi square test, **#**= one way ANOVA

*P* < 0.05 is considered statistically significant

There were significant differences in VAS scores at 4, 8, and 12 h with the highest median (range) values in group C 4(3–6) in comparison to group D 2(1–3) and group G 2(1–3) and (p-value < 0.001) (Table 2).

There were significant differences regarding postoperative pethidine consumption at 24 h, with the highest values in group C (p-value < 0.001) (Table 2).

As regards adverse effects (nausea, vomiting, headache, and dizziness), no differences between the three groups were found (Table 3).

**Table 3:** Adverse events

	Group C (*n* = 51)	Group D (*n* = 51)	Group G (*n* = 51)	*P* value *
Dizziness	3(5.9%)	5(9.8%)	5(9.8%)	0.71
Headache	3 (5.9%)	4(7.8%)	4(7.8%)	0.9
Nausea & vomiting	5(9.8%)	5(9.8%)	7(13.7%)	0.77

Data expressed as frequency (percentage), *= chi square test

p-value > 0.05 is considered statistically non-significant

## Discussion

This study assessed and compared the effects of low-dose IM dexmedetomidine (1 µg/kg) and oral gabapentin (600 mg) premedication on EA, pain severity, and hemodynamic changes after rhinoplasty in adults. The results showed that dexmedetomidine and gabapentin decreased the emergence agitation incidence, severity, midazolam requirement, VAS scores, and pethidine consumption in comparison to the control group, with lower values in the dexmedetomidine group; No significant differences between groups regarding hemodynamic parameters (HR & MAP), recovery time, or adverse events were recorded.

EA is a well-known complication after rhinoplasty, mostly due to posterior nasal bleeding, pain, and nasal block [3]. EA increases the risk of serious trauma, bleeding, tube and catheter removal, desaturation, and hemodynamic instability [2].

There are limited studies concerning adult EA. Although the prevalence of adult EA is less than child EA, it carries more risk of injury due to stronger uncontrolled behaviors [13].

Desflurane and sevoflurane increase the EA during recovery even without surgical intervention, as in MRI, due to their fast recovery and wash-out profiles [14, 15].

Different drugs have been used for prophylaxis and treatment of EA, such as midazolam, opioids (fentanyl, remifentanil), gabapentin, clonidine, and dexmedetomidine.

Dexmedetomidine has analgesic, anxiolytic, sedative, hypnotic, and anesthetic effects. At a low dose, it activates the central alpha-2 adrenergic receptors in locus coeruleus and induces NREM sleep-like (easily arousable and cooperative) status [16].

The ideal dose of dexmedetomidine for EA prevention has not been determined by the previous literature. Hypotension and bradycardia were reported with high doses and fast intravenous injections. So, the lowest dose according to the individual physical status and operation, in addition to different routes, should be used to prevent adverse events [4, 17, 18].

The premedication with intramuscular dexmedetomidine 1–1.2 µg/kg was not inferior to 1.5–2.4 µg/kg regarding EA; it provides sedation and promotes anesthesia without hemodynamic side effects in line with our study dose and results [5].

Dexmedetomidine reduced the incidence, duration, and severity of EA during recovery with fewer intraoperative movements [6].

Kim and his colleagues reported that emergence agitation incidence was lower in the dexmedetomidine group than in the control group (28% vs. 52%, *P* = 0.041) after nasal surgery, in line with our results [19].

In children, a meta-analysis reported that dexmedetomidine lowered the emergence agitation incidence and severity, in addition to reducing pain severity and analgesic requirements in ophthalmic, orthopedic, pediatric, and surgical procedures in line with our results [18, 20]. Dexmedetomidine reduced EA in children after general anesthesia for MRI with no hemodynamic or respiratory effects and promoted early hospital discharge [15].

Gabapentin prevents presynaptic calcium influx [7], increases GABA synthesis and concentration, and subsequently decreases excitatory neurotransmitter release and blocks stimulus progression in the brain and dorsal root ganglia [8]. It has anticonvulsant, anxiolytic, sedative, and analgesic effects [9]. Moreover, it decreased narcotic requirements and postoperative pain [10].

After reviewing the previous literature on preoperative gabapentin dose for EA and postoperative pain, the oral dose of 600 mg gabapentin was defined as the ideal dose [21] and used in this study.

In agreement with our results, premedication with 600 mg gabapentin reduced EA after rhinoplasty in adults. Although post-operative pain score, analgesic requirements, and adverse events were reduced, no significant differences were recorded [22], mostly due to different surgical techniques.

Azemati and his colleagues reported that gabapentin decreased the emergence agitation incidence and severity after mastectomy in adults with limited adverse effects; also, it reduced postoperative pain severity in line with our results [11].

Gabapentin decreased postoperative pain and narcotic requirements and increased postoperative sedation after breast, thyroid, abdominal, and spine surgeries [23].

In children, gabapentin reduced emergence agitation, postoperative pain, and analgesic needs after tonsillectomy with sevoflurane anesthesia [21]. The same findings were reported after an orthopedic clinical trial in children under combined general anesthesia and nerve block [24].

Finally, to the best of our knowledge and the limited literature on adult EA, this study considered the first one compared dexmedetomidine and gabapentin against control regarding the incidence, severity of EA, and postoperative pain in adults and showed the effectiveness of both drugs with lower values in dexmedetomidine without affecting hemodynamics (MAP and HR).

### Limitations

Lack of placebo in the other route than the study drug, small sample size, and single dose of study drugs.

## Conclusions

IM dexmedetomidine premedication was more efficient than gabapentin in the reduction of the emergence agitation incidence, severity, and postoperative pain scores after rhinoplasty in adults.

## Data Availability

No datasets were generated or analysed during the current study.

## Abbreviations

ASA-PS: American Society of Anesthesiologists- Physical status
EA: Emergence agitation
GABA: Gamma amino-butyric acid
HR: Heart rate
IM: Intramuscular
IV: Intravenous
MAP: Mean arterial blood pressure
NREM: Non-rapid eye movement
OR: Operating room
PACU: Post anesthesia care unit
RSAS: Riker sedation-agitation scale
VAS: Visual Analogue score
